# Phytomediated Silver Nanoparticles (AgNPs) Embellish Antioxidant Defense System, Ameliorating HLB-Diseased ‘Kinnow’ Mandarin Plants

**DOI:** 10.3390/molecules28052044

**Published:** 2023-02-22

**Authors:** Muhammad Umair Raza, Fozia Abasi, Muhammad Shahbaz, Maria Ehsan, Wajiha Seerat, Abida Akram, Naveed Iqbal Raja, Zia ur-Rehman Mashwani, Hammad Ul Hassan, Jarosław Proćków

**Affiliations:** 1 Department of Botany, PMAS-Arid Agriculture University, Rawalpindi 46300, Pakistan; 2 Department of Plant Biology, Institute of Environmental Biology, Wrocław University of Environmental and Life Sciences, Kożuchowska 5b, 51-631 Wrocław, Poland

**Keywords:** nanotechnology, citrus greening disease or Huanglongbing (HLB), AgNPs, monometallic, *Candidatus* Liberibacter asiaticus

## Abstract

Citrus production is harmed worldwide by yellow dragon disease, also known as Huanglongbing (HLB), or citrus greening. As a result, it has negative effects and a significant impact on the agro-industrial sector. There is still no viable biocompatible treatment for Huanglongbing, despite enormous efforts to combat this disease and decrease its detrimental effects on citrus production. Nowadays, green-synthesized nanoparticles are gaining attention for their use in controlling various crop diseases. This research is the first scientific approach to examine the potential of phylogenic silver nanoparticles (AgNPs) to restore the health of Huanglongbing-diseased ‘Kinnow’ mandarin plants in a biocompatible manner. AgNPs were synthesized using *Moringa oleifera* as a reducing, capping, and stabilizing agent and characterized using different characterization techniques, i.e., UV–visible spectroscopy with a maximum average peak at 418 nm, scanning electron microscopy (SEM) with a size of 74 nm, and energy-dispersive spectroscopy (EDX), which confirmed the presence of silver ions along with different elements, and Fourier transform infrared spectroscopy served to confirm different functional groups of elements. Exogenously, AgNPs at various concentrations, i.e., 25, 50, 75, and 100 mgL^−1^, were applied against Huanglongbing-diseased plants to evaluate the physiological, biochemical, and fruit parameters. The findings of the current study revealed that 75 mgL^−1^ AgNPs were most effective in boosting the plants’ physiological profiles, i.e., chl a, chl b, total chl, carotenoid content, MSI, and RWC up to 92.87%, 93.36%, 66.72%, 80.95%, 59.61%, and 79.55%, respectively; biochemical parameters, i.e., 75 mgL^−1^ concentration decreased the proline content by up to 40.98%, and increased the SSC, SOD, POD, CAT, TPC, and TFC content by 74.75%, 72.86%, 93.76%, 76.41%, 73.98%, and 92.85%, respectively; and fruit parameters, i.e., 75 mgL^−1^ concentration increased the average fruit weight, peel diameter, peel weight, juice weight, rag weight, juice pH, total soluble solids, and total sugarby up to 90.78%, 8.65%, 68.06%, 84.74%, 74.66%, 52.58%, 72.94%, and 69.69%, respectively. These findings enable us to develop the AgNP formulation as a potential citrus Huanglongbing disease management method.

## 1. Introduction

Genus *Citrus* belongs to the Rutaceae family. It is one of the most frequently cultivatedfruits in the world [1], with varieties growing in tropical, subtropical, and other climates, between latitudes of 35° N and 35° S [2]. Approximately 121 million tons of citrus are produced each year [3]. Citrus subsists in almost 150 genera and 1600 species [4], with the most commercially important being mandarin *Citrus reticulata* Blanco [5].Citrus fruits are eaten fresh, but are also processed for use in the cosmetics (e.g., fragrances, scrubs, and masques) and food (e.g., drinks, cakes, and candies) industries [6]. Citrus fruits are rich in vitamin C, a nutrient known to strengthen the immune system and ensure good heart health by effectively controlling cholesterol levels [7]. In Pakistan, citrus is grown mainly in Punjab and is one of the most widely exported fruits inthe country [8]. Almost 96% of the total citrus production is cultivated in Punjab [9]. The ‘Kinnow’ mandarin (*Citrus reticulata* Blanco), which is grown mainly in Sargodha and its adjacent areas, including the districts of Toba Tek Singh, Faisalabad, and Sahiwal, accounts for more than 80% of the citrus planted [10]. Currently, one of the most potent factors to reduce citrus yields includes a wide range of diseases caused by fungi, bacteria, nematodes, and viruses [11]. Huanglongbing (HLB), often known as citrus greening, is one of the deadliest bacterial invasive citrus diseases, having a global presence in more than 40 countries, and has been proven to be the most devastating for the citrus industry around the world [12,13]. HLB is widespread in most citrus-growing regions in Asia, Africa, and America [14]. HLB is caused by a fastidious phloem-limited Gram-negative uncultivable bacterium transmitted by vectors belonging to the α-subdivision of the Proteobacterium ‘*Candidatus* Liberibacter spp.’ [15]. HLB bacteria have three main strains, Asiaticus, Africanus, and Americanus, which have been distinguished based on environmental factors and insect vectors. ‘*Candidatus* Liberibacter asiaticus (CLas)’ is prevalent in most regions of Asia and America; it reduces the flow of vital nutrients in the phloem, affecting the health of citrus trees and their fruit quality [16,17]. Typical diagnostic symptoms of HLB can include leaf mottling, premature defoliation, severe yellowing of the veins and surrounding tissues, vein corking, small green fruits with aborted seeds, and twig dieback [18,19].

Huanglongbing disease has been identified as the main cause of the decline of citrus in Pakistan, resulting in significant losses to the citrus industry, especially in the Sargodha and Multan districts of Punjab province, together with the Malakand district of Khyber Pakhtunkhwa province [9]. The incidence of HLB disease ranges from 4.6%, as the lowest, recorded in Sahiwal, and the highest incidence of the disease among *C. sinensis* ‘Musambi’ of 26% in kotmomin [20], and for ‘Kinnow’ mandarin (*C. reticulata*), itwas 51.7% in non-core areas of Pakistan [21]. Currently, there is no cure for HLB infection, and no specific management measures have been developed to control HLB disease [22]. There are no good sources of HLB genetic resistance in the *Citrus* genus or its relatives [23,24]. Various strategies have beenused to hinder the propagation of HLB disease, including insecticides [25], antimicrobial agents [26], and injections of antibiotics into infected trees to reduce HLB symptoms [27]. However, the prospect of microbial resistance, as well as various indirect negative effects on human health, is a growing and pressing concern that restricts antibiotic use in the field [28].

**Figure 1:**
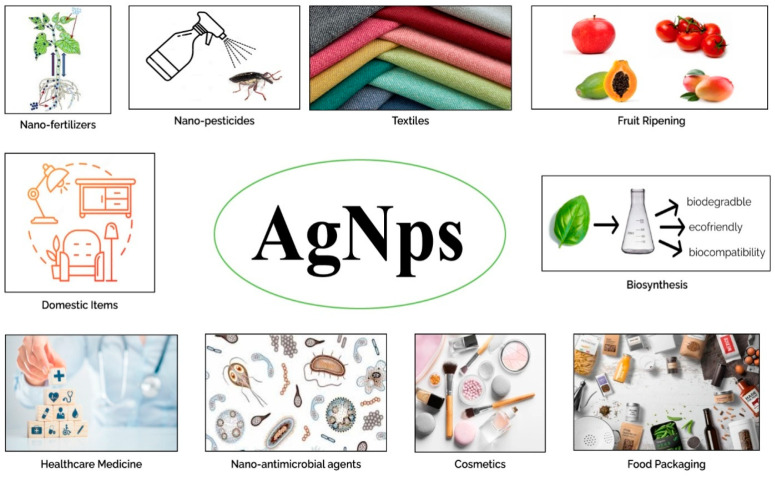
Schematic illustration of the potential of AgNPs. AgNPs can be used in household items, food packaging, textiles, cosmetics, the main components of medicines, and antiseptics in medical equipment [39,40,41]. AgNPs have been used to stimulate growth in plants [42], aid in fruit ripening [38], developed as fungicides to prevent fungal infections [39], and possess antidiabetic [43] and antibacterial potential [44,45], antiviral capabilities [43], and anticancer potential [44]. The activity of AgNPs against the causal agent of HLB ‘*Candidatus* Liberibacter asiaticus’ is also studied [46].

As a result, finding a long-term strategy to cure Huanglongbing disease with minimal side effects is critical. Nanotechnology concerns the formation of nanoparticles with sizes of 1–100 nm and plays a significant role in plant disease management [29]. It is the most advanced technology in thepresentera, having a wide range of applications in agriculture [30]. In this case, green nanotechnology has proven to be the most efficient and environmentally friendly technique against pathogen-based damage to diverse crops and fruits around the world, by reducing the use of agrochemicals, increasing the plant’s defense system against pathogen attack, improving nutrient uptake, and improving plant growth [31]. Green-synthesized silver nanoparticles (AgNPs) have emerged as effective antimicrobial and antioxidant agents to reduce the negative impacts of plant diseases in a variety of crops [32]. AgNPs produced from plant leaf extracts have been demonstrated to have excellent biocompatibility, bioavailability, and lower toxicity, and they are cost-effective, biodegradable, and eco-friendly [33]. Biogenic Ag nanomaterials’ increased methylglyoxal detoxification, antioxidant defense mechanisms, and tolerance to stress-induced ROS injury have been methodically explained in plants over the past ten years [34]. The plant extract is embellished with a variety of alkaloids, phenols, amines, and ketones that help to reduce agents and help to stabilize the capping in the fabrication of AgNPs [35]. AgNPs have different sizes, shapes, and biochemical functional characteristics that endow them with the ability to exerttherapeutic effects via a variety of molecular mechanisms [36]. A previous study revealed that silver nanoparticles have been used for disease management. Khan et al. [37] used phytosynthesized silver nanoparticles against plant parasitic nematode Meloidogyne incognita, and plant pathogens Ralstonia solanacearum and Fusarium oxysporum. Similarly, biogenic silver nanoparticles were applied against Stromatiniacepivora, which caused white rot disease in onion and garlic [38] (Figure 1).

The present study is confined to the synthesis of AgNPs using the extracts of *Moringa oleifera* (Moringaceae). Moringa or drumstick treeis highly rich in a variety of bioactive components, including phenolic acids, tannin, flavonoids, glucosinolates, alkaloids, terpenoids, saponins, protein, minerals, and vitamins, which contribute to its goodnutritional, nutraceutical, and medicinal profiles [47]. Antimicrobial [48], anticancer, antidiabetic, antioxidant, anti-atherosclerotic, antiproliferation, hepatoprotective, antiperoxidative, anti-inflammatory, and cardioprotective activities have been demonstrated in M. oleifera seeds [49].

To date, there has been no scientific research on the use of plant-mediated AgNPs to control Huanglongbing disease in *Citrus reticulata* ‘Kinnow’ mandarin plants. The presentstudy is the first to reveal that AgNPs affect physiological, biochemical, and antioxidant functions differently against the Huanglongbing-infected ‘Kinnow’ mandarin plant defense system. Exogenously, different concentrations of AgNPs were applied and used to identify the best effective concentration to treatHLB-infected ‘Kinnow’ mandarin plants.

## 2. Results and Discussion

### 2.1. Synthesis and Characterization of AgNPs

**Figure 2:**
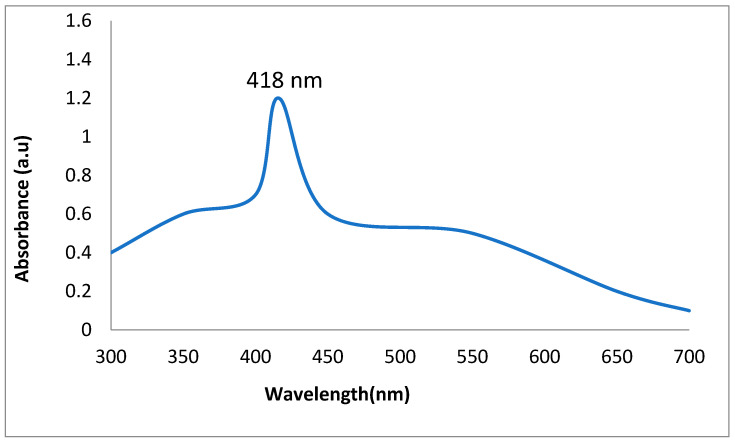
UV–vis spectroscopy of AgNPs.

The synthesis of AgNPs was validated by using the UV–visible spectrum between 200 and 800 nm. BiofabricatedAgNPs had characterization peaks in the range of 200 to 400 nm, according to the findings. However, the characterizing absorption peak was obtained at 418 nm, showing that plant-mediated AgNPs had surface plasmon resonance properties (Figure 2).

**Figure 3:**
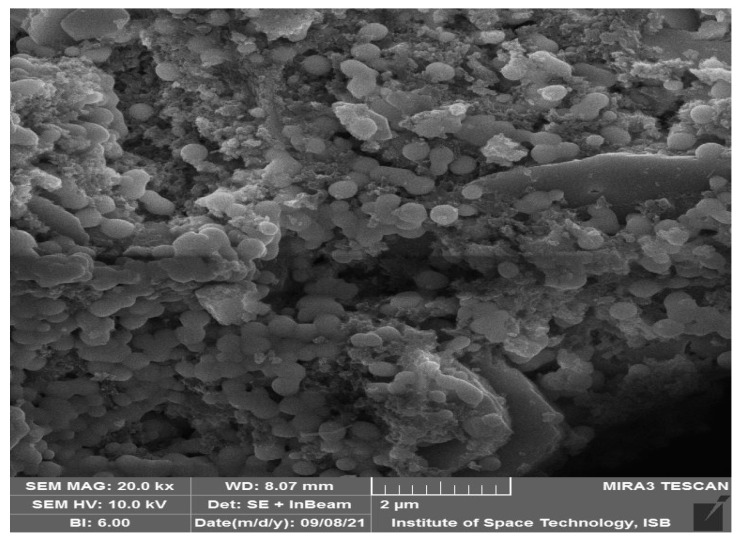
SEM of AgNPs.

SEM was used to performthe structural quantification of plant-mediated AgNPs. Green-synthesized AgNPs were spherical, cylindrical, or rectangular in shape, with an average size of 74 nm, as demonstrated in the SEM image (Figure 3). Some of these particles, however, were irregular and anisotropic in shape.

**Figure 4:**
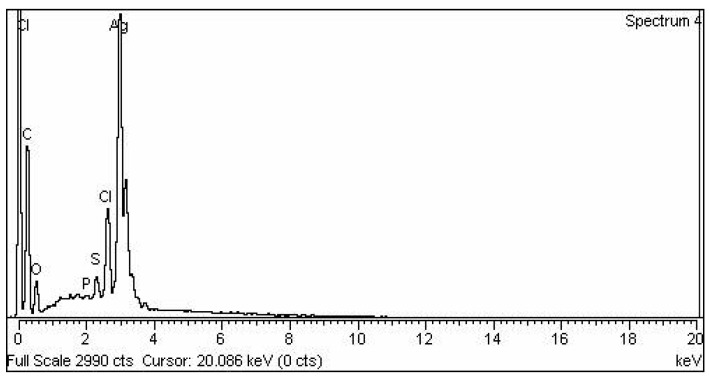
EDX of AgNPs.

Energy-dispersive X-ray analysis was performed to confirm the elemental makeup of the plant-mediated AgNPs. The purity and presence of AgNPs were confirmed by EDX spectroscopic analysis. The highest peaks of silver characterization ranged from 2.7 to 3.7 KeV (Figure 4).

**Figure 5:**
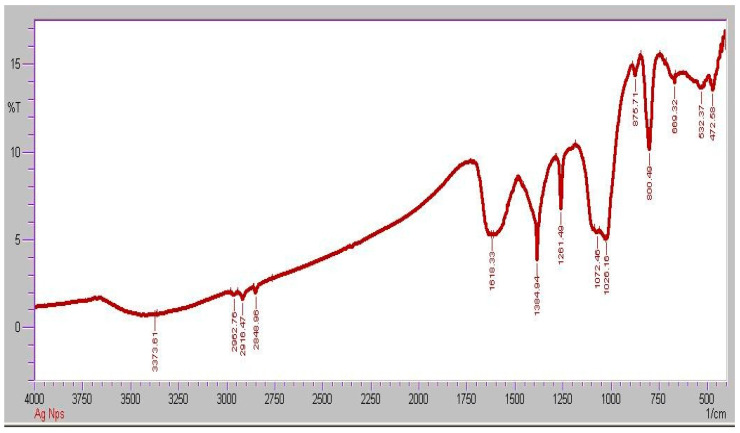
FTIR of AgNPs.

FTIR spectroscopy analysis was used to determine which organic biomolecules in the *Moringa oleifera* leaf extract were responsible for the fabrication of the spherical AgNPs (Figure 5). The following absorption peaks were found in the FTIR spectra of such phytofabricated AgNPs in the range of 450 to 4000 cm^−1^: 3419, 2922, 2370, 1734, 1637, 1508, 1458, 1366, 1261, 1097, 796, 619, and 453 cm^−1^. The source of these absorption peaks was determined to be the O–H stretching vibration (3419 cm^−1^) in alcohol, phenol, and flavonoid compounds [50], C–H stretching vibration (2922 cm^−1^) of aromatic compounds [51], C=O stretching vibration (2370, 1734 cm^−1^) for carbonyl compounds [52], C=C stretching vibration (1637 cm^−1^) of aromatic compounds [53], N–O stretching vibration (1508 cm^−1^) in aliphatic amines [54], C–H stretching vibration (1458 cm^−1^) in methylene moieties [55], C–O stretching vibration (1261, 1097 cm^−1^) in alcohols and phenols [56], C=C stretching vibration (796 cm^−1^) of aromatic compounds, and, finally [57], C–Br and C–Cl bonding vibration (619, 453 cm^−1^, respectively) in organic compounds. This confirmed that the organic compounds present in the *Moringa oleifera* leaf extract contributed to the reduction of Ag ions, resulting in the creation of AgNPs and the capping of the resulting nanostructures that provide stability, biocompatibility, and functionality in biological activity [58].

### 2.2. Physiological Parameters

**Figure 6:**
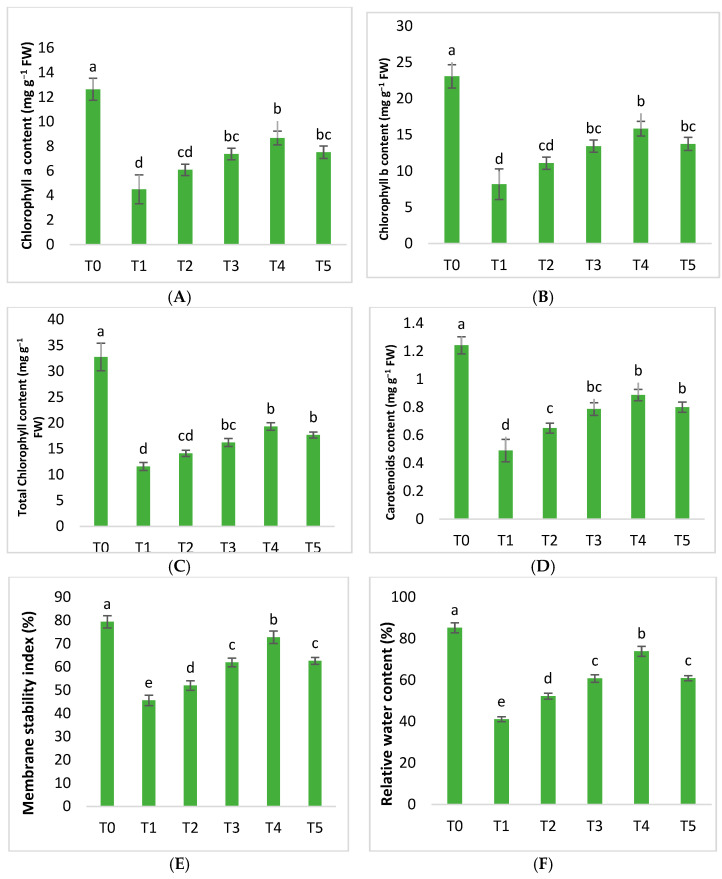
Differential effects of plant-based silver nanoparticles on (**A**) chl a, (**B**) chl b, (**C**) total chl, (**D**) carotenoid content, (**E**) RWC, and (**F**) MSI. (a mesns best than and b means best in second order and so on).

The potential of the green AgNPs against Huanglongbing-diseased citrus mandarins was examined physiologically. In the current findings, chl a, chl b, total chl, and carotenoids were reduced by 64.42%, 64.47%, 64.61%, and 60.58%, respectively, due to HLB disease. However, the application of AgNPs significantly increased the chl and carotenoids. AgNPs improved chlorophyll along with carotenoids, with an increase in concentration from 25 mgL^−1^ up to 75 mgL^−1^ compared to untreated diseased citrus plants. AgNPs at 75 mgL^−1^ exhibited, surprisingly, a significant increase in chl a, chl b, total chl, and carotenoid content by 92.87%, 93.36%, 66.72%, and 80.95%, respectively. The results demonstrated that AgNPs at 100 mgL^−1^ enhanced the above values to 67.21%, 67.64%, 52.54%, and 63.26%, compared to untreated plants (Figure 6).

In plants, the amount of chlorophyll and carotenoids determines the rate of photosynthesis. Chlorophyll helps to absorb sunlight for plants, asdo carotenoids; along with absorbing light energy, carotenoids provide photoprotection by nonphotochemical quenching. Plants’ photosynthetic capacity can be damaged by phytopathogenic bacteria, which cause necrosis and a drop in chlorophyll. Biotic stress inflicts significant oxidative damage, and ROS generation causes the photosynthetic machinery to be destroyed, resulting in plant mortality [59,60]. When plants are attacked by numerous pathogens, preserving their chlorophyll and carotenoid concentrations is critical, since this allows the plants to continue performing the photosynthetic process. The level of chlorophyll and carotenoid in mandarin plants is reduced due to Huanglongbing, as the current study suggests. Foliar treatments withAgNPs considerably improved the chlorophyll and carotenoid concentrations in HLB-infected plants (Figure 6). Plants that were treated with the dosage of silver nanoparticles set at 75 mgL^−1^ had the highest chlorophyll and carotenoid content. The amounts of chlorophyll and carotenoids decreased when the concentration of the AgNPs increased to 100 mgL^−1^.

In plants, the effects of AgNPs under biotic stress are not well understood. The current findings, which show a considerable improvement in the chlorophyll and carotenoid content, are consistent with those of Sadak et al. [61] and Gupta et al. [62], who found that AgNPs enhanced the chlorophyll content of *Oryza sativa* L. Furthermore, Khalofah et al. [63] observed that AgNPs increased the carotenoid and chlorophyll content in the leaves of *Linum usitatissimum* L.

In addition, an earlier study revealed that silver could speed up chlorophyll biosynthesis by easing the electron transport chain and the respiratory process. As a result, a rise in chlorophyll content in HLB-infected plants treated with AgNPs could help to restore the photosynthetic machinery and thus growth qualities. Furthermore, devastating effects on the stroma and grana lamellae are avoided by silver nanoparticles; AgNPs also help chloroplasts to protect chloroplast enzymes and regulate soil bacterial diversity, thus speeding up the photosynthetic machinery’s biosynthesis [64].

Furthermore, the membrane stability index (MSI) and the relative water content (RWC) are important variables in the appropriate physiological functioning of plants. AgNPs had various effects on diseased citrus plants. MSI and RWC levels were reduced by 42.60% and 51.71%, compared to healthy mandarins. However, the findings of the current study demonstrated that AgNPs steadily improved both of these respective parameters.

As the concentration of silver nanoparticles increased from 25 to 50 to 75 mgL^−1^, an improvement was noticed in the diseased plants. In particular, 25 mgL^−1^ AgNPs boosted the MSI and RWC content to 14% and 26.94%, respectively. The best results were notedat 75 mgL^−1^ of AgNPs, increasing the MSI and RWC content to 59.61% and 79.55%, following Huanglongbing-untreated mandarins. However, both of these amounts declined as the concentration of silver nanoparticles increased to 100 mgL^−1^ (Figure 6).

Plant MSI and RWC are severely reduced by biotic stressors. A low water supply causes a decrease in turgor pressure in plants, limiting cell expansion and causing morphological, physiological, and biochemical problems. The structural integrity of the plasma membrane is distorted due to the production of reactive oxygen species and causes electrolyte leakage inflicted on the cell. The amount of RWC in plants is determined by their water absorption and transpiration rate [65]. The findings demonstrated that, compared to unaffected control plants, biotic stress considerably decreased the relative water and membrane stability content in Huanglongbing-affected plants. On the other hand, foliar applications of plant-based AgNPs increased RWC and MSI (Figure 6).

Silver nanoparticles have been found to increase antioxidant defenses, lower ROS levels, and improve RWC and cell membrane integrity among plants [66]. Further, the results of the current study are in line with Shahbaz et al. [67], who found thatsilver nanostructures improved RWC while maintaining plasma membrane integrity under stress conditions [68]. The decrease in chlorophyll and carotenoid content in Huanglongbing-diseased plants applied with AgNPs at 100 mgL^−1^ could be attributed to a variety of factors, including ROS formation, inflicting severe oxidative damage to the plant.

### 2.3. Biochemical Parameters

**Figure 7:**
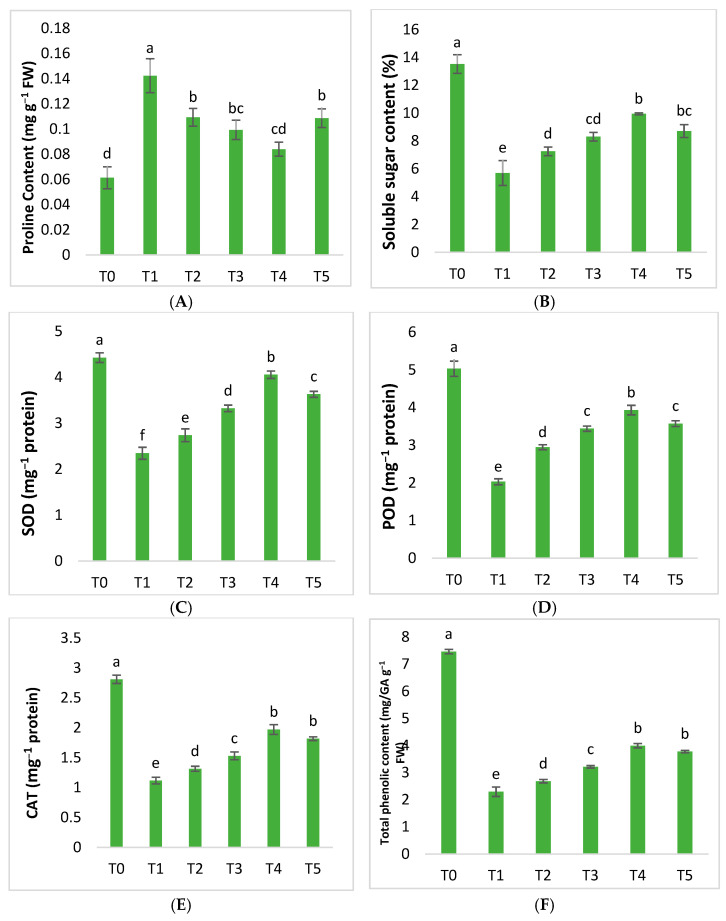

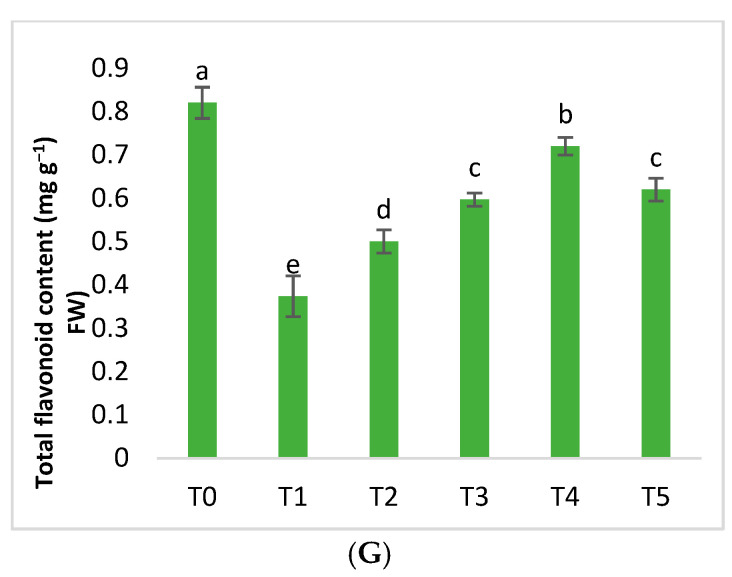
Differential effects of AgNP treatments on the following content: (**A**) proline, (**B**) SSC, (**C**) SOD, (**D**) POD, (**E**) CAT, (**F**) TPC, and (**G**) TFC.

Infected ‘Kinnow’ mandarin plants produce more proline as a result of HLB disease. The proline content increased by 132.06% compared to healthy ones. However, our findings revealed that plant-based AgNPs showed various effects on proline content formation. Exogenously applied silver nanoparticles at 25 mgL^−1^ reduced proline by 23.18% in diseased citrus plants. The application of 50 mgL^−1^ AgNPs further helped to decrease the respective content by 30.21% as compared to untreated plants. Silver nanoparticles at a75 mgL^−1^ concentration proved to be the best applied formulationof AgNPs, which decreased the proline content by up to 40.98%. However, the content was found to have increased with an AgNP concentration at 100 mgL^−1^ (Figure 7).

Sugars play a crucial role as osmolytes in helping plants to defend themselves from biotic stress. Our results show that the soluble sugar content of plants is significantly affected by the HLB disease. The findings showed that compared to healthy plants, the level of SSC in diseased plants was reduced by 57.93%. The application of AgNPs dramatically improved SSC in diseased plants compared to healthy ones. The concentration of silver nanoparticles at 25, 50, and 75 mgL^−1^ helped to enhance the amounts of SSC. The application of AgNPs at 75 mgL^−1^ produced the best results, increasing SSC by 74.75% compared to plants infected with Huanglongbing that were not treated. The best results were obtained withAgNPs at 75 mgL^−1^, which increased SSC by 74.75%. However, the SSC amounts decreased as the AgNP concentration increased to 100 mgL^−1^ (Figure 7).

In plants, proline production is massively boosted as a result of stress being inflicted upon plants by their surroundings. Proline generation is essential to restore normal stress levels. Proline helps to protect plants against oxidative damage and maintains a healthy osmotic environment. Furthermore, it also protects proteins against denaturation when exposed to harsh circumstances [68].

Similarly, soluble sugars play an important role in the defense mechanisms of plants. Sugars are the basic substrate for plant defense responses, providing structural material and energy. Sugar content also affects the plant immune system by acting as a signal molecule that interacts with hormone signaling [69]. The results of the study show that the proline content is higher in plants with HLB disease than in healthy plants, as, under stress, plants produce proline to deal with the detrimental effects of biotic stressors. However, the findings of the current study revealed that AgNPs mediated by plant extracts decreased the proline levels in diseased mandarin plants. The results are consistent with Fimognari et al. [70] and Hadwan et al. [71], finding that the exogenous application of AgNPs decreased proline levels, demonstrating a stress reduction. HLB-diseased ‘Kinnow’ plants had a reduced amount of total sugars, according to the findings; however, the applied AgNPs raised the level of SSC. The findings corroborate those of Salih et al. [72], who found that silver nanoparticles improved the total sugar synthesis in tomato plants affected by stress circumstances.

Varied amounts of plant-mediated AgNPs considerably boosted the performance of enzymatic and non-enzymatic antioxidants such as superoxide dismutase, peroxidase, catalase, total phenolics, and flavonoid content. However, HLB disease reduced the above-mentioned content of SOD, POD, CAT, TPC, and TFC by 46.98%, 59.66%, 60.26%, 69.25%, and 54.47%, respectively (Figure 7). Plant-mediated AgNPs had various effects on diseased citrus plants, altering antioxidant activity to ameliorate the negative consequences. AgNPs at 50 mgL^−1^ increased this potential content by 41.61%, 69.45%, 37.01%, 40.11%, and 59.82%, respectively (Figure 7). AgNPs at 75 mgL^−1^ were shown to improve the antioxidant defense of Huanglongbing-infected plants by upregulating the respective activities, such as SOD, POD, CAT, TPC, and TFC content. They increased by 72.86%, 93.76%, 76.41%, 73.98%, and 92.85%, respectively. However, the results demonstrated that these activities decreased as the AgNP concentration increased to 100 mgL^−1^ (Figure 7).

Living organisms rely on their immune systems to protect them from diseases that can be deadly. Immune-specified disorders are quite common among animals, but are rarely investigated in plants. HLB disease falls into this category, as it attacks the antioxidant defense system of citrus plants [73]. In this respective work, the results demonstrated that HLB drastically reduced the levels of enzymes that help in defense, i.e., CAT and POD [74]. Exogenous foliar treatments with green-synthesized AgNPs increased the levels of defense-related enzymes in diseased plants compared to diseased mandarins (Figure 7). According to the findings, the use of AgNPs at 75 mgL^−1^ proved ideal in increasing antioxidant enzymes; the respective findings are consistent with Fang et al. [75], whofoundthat silver nanoparticles boosted the levels of enzymes. In plants, limited study has beendone to implement AgNPs against biotic stressors. However, it has been observed that the application of AgNPs increased the amount of SOD and CAT enzymes in stressed rice plant seedlings, increasing their capacity to withstand stress [76]. Similar to this, numerous other studies show that the use of silver nanoparticles in stressed tomato plants increased the activity of POD and SOD [77]. Our results are based on those of Mirmoeini et al. [78], who found that plants have an antioxidant defense response that is mediated by silver. At the same time, the study findings revealed that 100 mgL^−1^ silver nanoparticles reduced the amount of antioxidant defense enzymes; this may be due to the toxicological effects of a high concentration of AgNPs, which could have contributed to an increase in silver, because silver at high levels functions as apro-oxidant and has dramatic impacts on plants, according to several published research works, such as Qian et al. [79].

In plants, biotic or abiotic stressors disrupt the electron transport pathway, which causes the generation of ROS, being powerful oxidizing agents that harm plant cells [80]. Subsequently, plants respondusing the enzymatic and non-enzymatic chemicals as a defensive mechanism against the harmful effects of ROS that neutralize their effects and protect cells [81]. Plants produce non-enzymatic antioxidants such as TFC and TPC as an instinctive response to stress [82]. Many studies have found that the use of various nanoparticles can cause the creation of antioxidant chemicals, which can help plants to resist pathogens [83]. In infected plants, non-enzymatic chemicals were found to be lower than in healthy control plants, according to our findings). This is because HLB infections inflict a severe, devastating impact on the antioxidant defense systems of plants. However, compared to untreated diseased trees, the application of AgNPs increased the production of TFC and TPC in HLB-diseased plants. The current findings are consistent with those of Chung et al. [84], who found that silver nanoparticles altered the metabolic profile in *Cucumis anguria* plants under biotic stress by increasing the defense systems. However, Raigond et al. [85] found that ZnNPs increased TPC levels in potato plants, supporting our findings. The excellent antioxidant capacity of AgNPs could be attributed to the numerous phytochemical functional groups, which enable the capping and stabilization of nanoparticles, thus contributing to the enzymatic and non-enzymatic attributes of plants [66].

### 2.4. Fruit Quality Parameters

**Figure 8:**
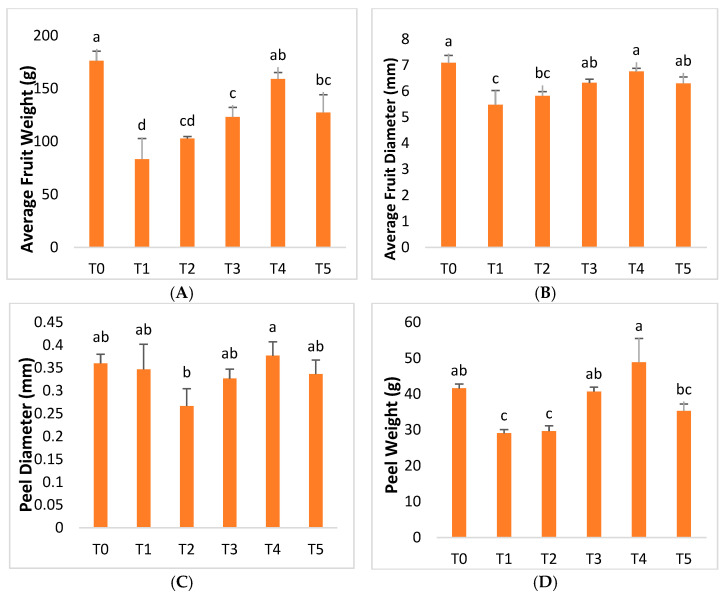

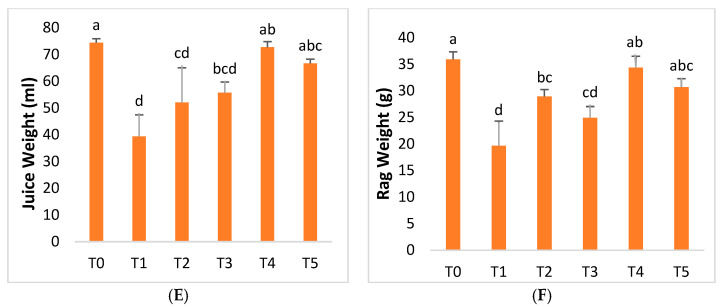
Differential effects of treatments of AgNPs on (**A**) average fruit weight, (**B**) average fruit diameter, (**C**) peel diameter, (**D**) peel weight, (**E**) juice weight, and (**F**) rag weight.

The findings revealed that the average fruit weight decreased by 52.72% in infected plants. However, compared to untreated ‘Kinnow’ mandarin plants, the treatment of AgNPs significantly increased the average fruit weight in diseased plants. The optimum results were obtained with an AgNPs concentration at 75 mgL^−1^, which increased the average fruit weight by up to 90.78% compared to Huanglongbing-untreated plants. However, the average weight of the fruit decreased as the AgNPs concentration rose to 100 mgL^−1^ (Figure 8). Furthermore, according to the research, the average diameter of the fruits in infected plants was 22.75% lower than in healthy plants. However, the average fruit diameter was increased to a considerable value after the application of AgNPs. The study revealed that AgNPs at 25 mgL^−1^ increased the mean diameter of the fruit by 6.26%, an increase of 15.39% was recorded at 50 mgL^−1^ of AgNPs, and AgNPs at 75 mgL^−1^ generated the optimal results, increasing by 23.37% compared to diseased plants. However, the mean diameter measurement of the fruit decreased to 14.97% as the AgNP concentration was increased to 100 mgL^−1^ (Figure 8). Compared to healthy plants, Huanglongbing disease reduced the diameter of the peel, the weight of the peel, the weight of the juice, and the weight of the rag by 3.70%, 30.08%, 47.08%, and 45.23%, respectively (Figure 8). AgNPs at 75 mgL^−1^ were shown to be the ideal formulation, with 8.65%, 68.06%, 84.74%, and 74.66% increases in peel diameter, peel weight, juice weight, and rag weight, respectively. However, a decrease was recorded in the measurements of further AgNP treatments (Figure 8). Fruits are a very important attribute that determines the productivity of fruit crops. Fruits appear on the plant after a certain amount of time of plant growth. Hence, any factor affecting the plant will automatically impact the quality and productivity of the fruit. Fruits are affected by different diseases in unique and diverse ways, ranging from physical to biochemical characteristics [86]. Citrus greening disease, or HLB, has serious impacts on diseased plants, and these impacts are variably observed in the average fruit weight, fruit diameter, peel diameter, peel weight, rag, and juice weight. The investigation’s findings demonstrated that Huanglongbing significantly decreased the average weight compared to healthy citrus plants, thus having variable impacts on the fruit diameter, peel diameter, peel weight, rag weight, and juice weight [87].

Exogenous foliar treatments with AgNPs enhanced the levels of relative attributes described in plants infected with Huanglongbing (Figure 8). According to this respective research, the use of AgNPs at a dose of 75 mgL^−1^ produced very promising results. The findings of this study are similar to those of Nejatzadeh et al. [88], who discovered that silver nanoparticles increased the germination speed, plant height, and stem length under salinity stress.

**Figure 9:**
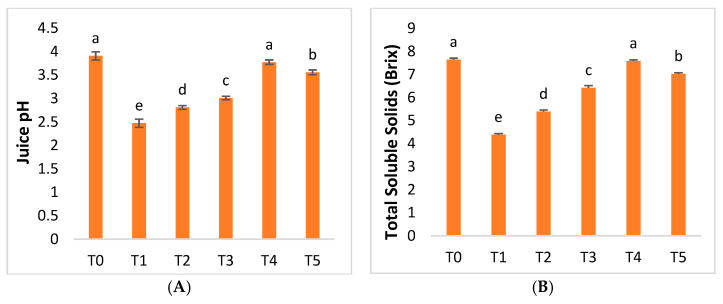

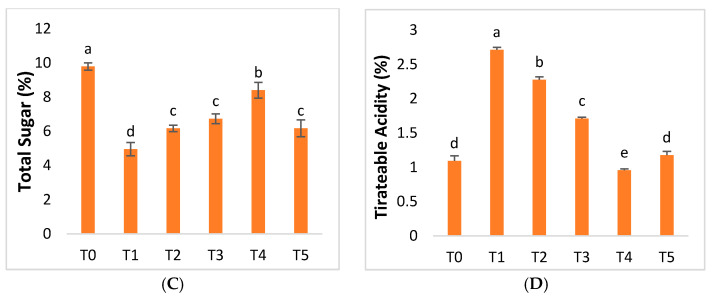
Differential effects of AgNP treatments on (**A**) juice pH, (**B**) total soluble solids, (**C**) total sugar, and (**D**) titratable acidity (TA).

HLB disease had differential effects on juice pH, total soluble solids, and total sugar in mandarin plants. Juice pH, TSS, and TS were reduced by 36.67%, 42.60%, and 49.38%, respectively, due to HLB disease (Figure 9). AgNPs at 75 mgL^−1^ were shown to be the most optimal formulation, with improvements in juice pH, total soluble solids, and total sugar of 52.58%, 72.94%, and 69.69%, respectively. The AgNP concentration, when increased above this level, led toa decrease (Figure 9). Infected ‘Kinnow’ mandarin plants have more titratable acidity (TA) as a result of HLB disease. Titratable acidity (TA) increased by 148.17%compared to healthy plants. Our findings revealed that plant-based AgNPs showed diverse effects on titratable acidity (TA) production. The exogenous application of AgNPs proved to be effective in decreasing TA. AgNPs at 25, 50, and 75 mgL^−1^ reduced TA by 16.09%, 36.97%, and 64.74%. Hence, AgNPs at 75 mgL^−1^ proved to be the optimal concentration. However, the titratable acidity increased when the AgNP concentration also increased (Figure 9).

The biochemical quality parameters of the fruit, also known as internal quality parameters, including acids, soluble solids, and sugars, help us to accurately calculate the maturity index, helping us to determinethe ripe fruit of the ‘Kinnow’ mandarin plant to its maximum potential for these biochemical characteristics [89,90]. Before harvesting, citrus maturity standards, such as the juice content, soluble solids, and acid ratio, are applied in the modern world, with variations according to citrus fruits and exporting market norms [91,92]. HLB disease, or citrus greening disease, has a significant influence on HLB-infected ‘Kinnow’ mandarin plants, with pH, TA, TSS, and TS levels varyingin this study. Two related concepts that address acidity in food analysis are TA and pH. Compared to healthy citrus plants, the pH level of the juice of the HLB-infected ‘Kinnow’ plant was significantly lower [93]. Exogenous foliar sprays of green-synthesized AgNPs improved the pH levels in HLB-infected plants, thus helping the plants to achieve abetter flavor and texture. Using an AgNP dose of 75 mgL^−1^ yielded very promising results, according to this study (Figure 9). The findings of this work are similar to those of Srivastava et al. [94], who discovered that nanostructured silver particles improved the pH levels during the catalytic degradation of azo dyes through the electron relay effect. TA predicts the ways in which organic acids affect the flavor of food. Strong acids are completely dissociated; on the other hand, food acids are only partially ionized. Although the overall acid content affects some dietary characteristics, others are only affected by the ionized fraction of the acid molecules. When intrinsic acids are titrated with a reference base, the TA of the food is evaluated to establish its overall acid content [95]. Compared to healthy citrus plants, the titratable acidity (TA) of HLB-infected ‘Kinnow’ plants was much higher [77]. Exogenous foliar sprays of green-synthesized AgNPs decreased thetitratable acidity (TA) in HLB-infected plants, aiding plants with better flavor. According to this study, the use of 75 mgL^−1^ AgNPs was the most effective and produced the best results. The findings of this study are similar to those of Alidoust et al. [96], who found that nanoparticles decreased the growth of TA levels inrice plants.

Total soluble solids (TSS) are also an essential indicator of fruit quality, accounting for 10–20% of the fresh weight of the fruit. The TSS value has an impact on the taste of the fruit because it indicates the level of sweetness of the fruit. As the fruit grows, it becomes sweeter and less acidic. Therefore, TSS plays an important role in fruit maturation, contributing to the economic benefits of the trade of fruits [97]. Compared to healthy citrus plants, the TSS level of HLB-infected ‘Kinnow’ mandarin plants was much lower [80]. Exogenous foliar sprays of green-synthesized AgNPs increased the TSS levels in HLB-infected plants, helping plants to taste better (Figure 9). According to this study, the use of an AgNP dose of 75 mgL^−1^ produced highly promising results. The findings of this study are similar to those of Faghihi et al. [98], who found that asilver nanocomposite with grapefruit peel improved the TSS levels and thus the nutritional value of the cucumber after harvest.

All monosaccharides and disaccharides found in food, regardless of their source, constitute total sugars (TS). The term “sugar” normally refers to sucrose (table sugar), although it can also refer to all sugars [99]. Sugars are the main source of energy and structural material for plant defense responses, and may also act as signal molecules that interact with the hormone signaling network of the plant immune system [100]. The TS level of HLB-infected ‘Kinnow’ mandarin plants was considerably lower than that of healthy citrus plants [101]. Exogenous foliar sprays of green-synthesized AgNPs increased the TS levels in ‘Kinnow’ plants infected with HLB, helping them to obtain higher taste quality (Figure 9). Using AgNPs at a dose of 75 mgL^−1^ yielded highly effective results, according to this study. The findings of this study are comparable to those of Faghihi.

## 3. Materials and Methods

For the experiment, 8-year-old ‘Kinnow’ trees (*Citrus reticulata*) were selected in Chak no 77 SB Sargodha, Pakistan. Chak no 77 is located in the Sargodha district, Punjab province, Pakistan; its geographical coordinates are latitude, 32°03′40.6″ N and longitude, 72°52′51.5″ E, with climatic conditions comprising summers that are hot, humid, and short, while winters are short, cool, and dry. The ‘Kinnow’ mandarin trees with severe symptoms were distinguished and marked with blue ribbons along with specific digit codes assigned to different branches to analyze the impacts imparted by the application of AgNPs. Leaves showing symptoms of HLB were collected from 25 different trees to confirm the presence of the causative agent ‘*Candidatus* Liberibacter asiaticus’ (CLas) by adopting the conventional PCR method with accession number (https://www.ncbi.nlm.nih.gov, and accessed on 13 December 2021).

### 3.1. AgNPs Synthesis

#### 3.1.1. Preparation of Plant Extract

Fresh and healthy leaves were collected from the main campus of University Rawalpindi, Punjab, Pakistan; they were washed with distilled water and we added 20 g fresh leaves into a 500 mL beaker containing 200 mL distilled water. Leaves were heated at 80 °C for 25 min using a hot plate. After heating, the solution was filtered two to three times to obtain apure and transparent Moringa oleifera leaf extract. The required amount of leaf extract was used to synthesize silver nanoparticles and the remaining was stored at 4 °C for further use [102].

#### 3.1.2. Biofabrication of AgNPs

**Figure 10:**
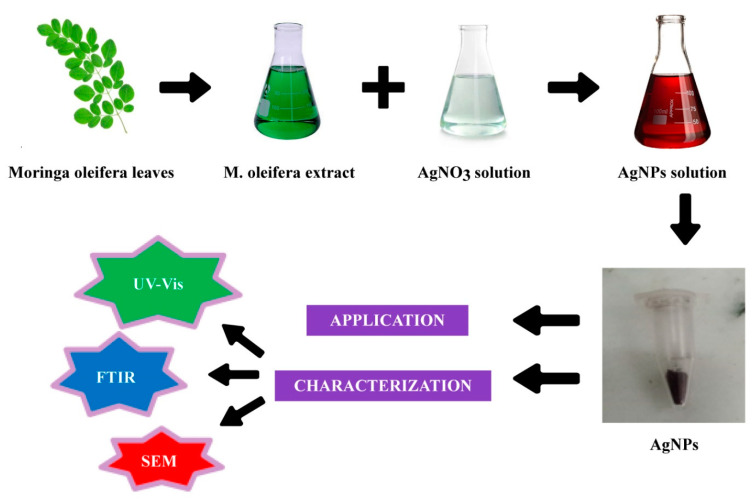
Methodology of green synthesis of silver nanoparticles.

Approximately 50 mL of *Moringa oleifera* aqueous leaf extract was poured dropwise into 450 mL of silver nitrate solution (5 mM) and the mixture was stirred continuously for 4 h at room temperature for complete AgNP biosynthesis. Color changes of reaction mixtures were closely monitored to confirm nanoparticle formation; AgNP production was characterized by a dark brown color of the reaction solution [103]. After the formation of AgNPs, the solution was stored in the dark at room temperature to prevent the agglomeration of AgNPs in the solution. Subsequently, the solution was centrifuged at 10,000 rpm for 15 min at 8 °C to collect pure AgNPs, the supernatant was collected, and purified AgNPs were oven-dried at 80 °C overnight for UV–vis analysis [104,105] (Figure 10).

### 3.2. Characterization of Nanoparticles

Characterization of AgNPs was achieved using UV–visible spectrometry, X-ray diffraction (XRD), scanning electron microscopy (SEM), and Fourier transform infrared (FTIR).

### 3.3. Experimental Plan and Application of Biogenic AgNPs

**Figure 11:**
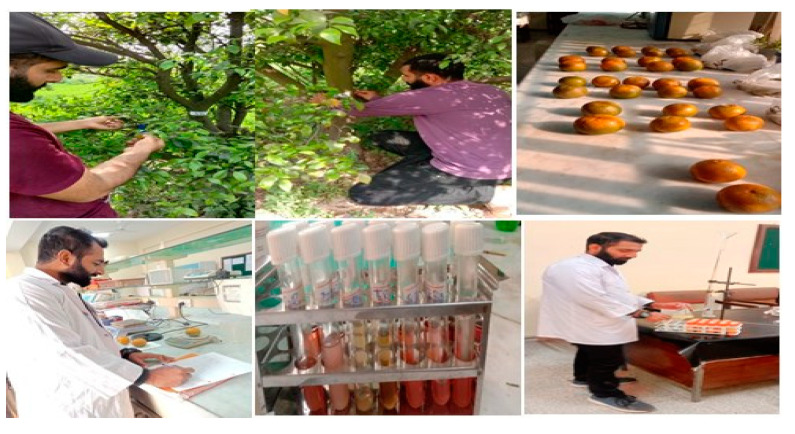
Showing the sample collection and evaluation of fruit parameters.

To evaluate the effects of different doses of biofabricated AgNPs, 20 diseased trees were used for exposure to the AgNPs. Six distinct treatments, three replicates, and various amounts of biogenic silver nanoparticles were applied topically to ‘Kinnow’ mandarins that had been exposed to HLB. The foliar treatments were applied using a sprayer (Hand Sprayer AP-20P, Jiaodian Technology Co., Wuhan, China). AgNPs were sprayed at 5:00 and 10:00 in the morning to ensure the gaseous exchange process through the stomata. Before the flowering stage in February 2021, all treatments were applied exogenously twice at 14-day intervals. In Table 1, a complete treatment plan is provided (Figure 11).

### 3.4. Physiological Parameters

#### 3.4.1. Chlorophyll and Carotenoid Content

At wavelengths of 480, 645, 652, and 663 nm, absorbance was observed using a spectrophotometer (Model U-2900 Sr. 26E82-018 Hitachi High-Teck Global Jp) against 80% solvent acetone as a blank [106]. The following formula was used to determine the chlorophyll content [107]:

Chl a (mgL^−1^) = 12.7 (A663) − 2.69 (A645)

Chl b (mgL^−1^) = 22.9 (A645) − 4.68 (A663)

Total chl (mgL^−1^) = 20.2 (A645) + 8.02 (A663)

Carotenoid = [A480 + (0.114 (A663) − (0.638-A645)] × V/1000 × W

A is the absorbance measured at the respective wavelength.

#### 3.4.2. Relative Water Content

To assess RWC, the method proposed by Hussain et al. [108] was followed.

RWC = (fresh weight − dry weight)/(saturated weight − dry weight) × 100

#### 3.4.3. Membrane Stability Index

The protocol of Karami et al. [109] was followed and the following formula was used to compute the MSI:

MSI = [1 − (C1/C2)] × 100

#### 3.4.4. Peroxidase, Superoxide Dismutase, and Catalase Activity

The peroxidase dismutase (POD) activity was determined using a spectrophotometer (Shimadzu, UV micro-1240) as described by Chandra et al. [110]. The ability of superoxide dismutase (SOD) to resist a reduction in NBT was investigated following the method published by Marslin et al. [111]. The method described by Kasote et al. [112] was used to test the catalase (CAT) activity of treated and untreated citrus plants.

#### 3.4.5. Total Phenolic Content (TPC)

The Folin–Ciocalteu reagent method proposed by Vennila et al. [113] was followed to determine the total phenolic content.

#### 3.4.6. Total Flavonoid Content (TFC)

The aluminum chloride colorimetric procedure was adopted for the determination of TFC as proposed by Mattos et al. [114].

#### 3.4.7. Proline Content and Sugar Content

The proline content of the treated and untreated leaves of the ‘Kinnow’ mandarin plants was measured at 520 nm following the method published by Kunwar et al. [115]. For sugar content, absorbance was observed at a 490 nm wavelength, adopting the procedure of Thompson et al. [116].

$$Soluble\;Sugar=\frac{\;Sample\;absorbance\times dilution\;factor\times K\;val}{Weight\;of\;Fresh\;Plant\;Tissue}$$

#### 3.4.8. Fruit Weight, Fruit Diameter, Peel Diameter, and Peel Weight

For the diameter, 36 ‘Kinnow’ mandarin fruit samples were randomly selected against specific treatments that comprised their respective replicates. Using an electronic weighing balance (Digital Electronic Lab Weighing Balance Scale—5000 g × 0.1 g), the average weight of each fruit was determined. The diameter of the fruit was measured using a manual Vernier caliper in millimeters. The fruits were thoroughly washed, dried, and peeled. The diameter of the peel (thickness) was measured with a Vernier caliper (mm). Subsequently, the peel weight was also measured using an electronic weighing balance [117].

#### 3.4.9. Juice Weight, Rag Weight, and Juice pH

The samples of the ‘Kinnow’ mandarin fruits were peeled and the juice was extracted in a beaker and poured into a measuring cylinder with readings. Therefore, the weight of the juice was measured (mL) and the remaining rag was weighed using an electronic weighing balance (Digital Electronic Lab Weighing Balance Scale—5000 g × 0.1 g) [118]. The pH of the juice was measured using a digital pH meter (HI-98127) [65].

#### 3.4.10. Total Soluble Solids and Titratable Acidity

The TSS was recorded using a digital spectrometer (AtagoPAL-3 refractometer). Juice samples of ‘Kinnow’ mandarin fruits were placed in the refractometer detector three times and the average value of each sample was recordedafter cleaning the refractometer detector with distilled water. TSS measurements were taken to obtain the percent Brix value, which indicated the sweetness level of the sample [119]. Titratable acidity (TA) was recorded taking into account the protocol used by Tyl et al. [120].

### 3.5. Statistical Analysis

In the factorial experimental arrangement of the treatments, all experiments were laid out. Each treatment was repeated three times. Analysis of variance (ANOVA) was applied to the data obtained using Microsoft Excel 2007 and STATISTICS 10 software.

## 4. Conclusions

The present research study demonstrates that treating Huanglongbing-diseased ‘Kinnow’ plants with green silver nanoparticles (AgNPs) is a biocompatible and environmentally benign method. *Moringa oleifera* plant extract was used as a reducing, capping, and stabilizing agent in the environmentally friendly synthesis of AgNPs in the current study. The AgNPs produced by green synthesis were spherical, cylindrical, or rectangular according to scanning electron microscopy. The increases in photosynthetic capacity, total sugars, and other fruit quality indices were more pronounced in plants infected with Huanglongbing when AgNPs were used at 75 mgL^−1^. Furthermore, cured plants showed a significant increase in antioxidants, and a significant reduction in titratable acidity (TA) and proline content was detected, highlighting the role of green AgNPs in reducing the devastating impacts of HLB. To summarize, the remarkable antibacterial potential discovered for these AgNPs at a concentration of 75 mgL^−1^ with a short exposure time may provide a viable solution to a disease that has yet to be eradicated. Overall, the study findings help citrus growers to achieve the better control Huanglongbing through management and treatment with a revolutionary control strategy and the introduction of the first biocompatible drug. The huge potential applications of phyto-nanotechnology in agriculture make it an essential study topic that demands the attention of plant scientists. To fully understand the relationship with silver nanoparticles, other factors that have an impact on citrus trees, and the responses of plants as a result, extensive scientific investigation is required. To explore the precise molecular activity of silver nanoparticles under in vivo circumstances, plant biologists, physiologists, pathologists, and nanotechnologists must work together. This will significantly affect the control of HLB disease in citrus and help to safeguard citrus production around the world.

## Figures and Tables

**Table 1:** AgNPs treatment plan.

Treatment	Conditions
T0	Control (healthy plants)
T1	Diseased (without AgNPs)
T2	Diseased + 25 mgL^−1^ AgNPs
T3	Diseased + 50 mgL^−1^ AgNPs
T4	Diseased + 75 mgL^−1^ AgNPs
T5	Diseased + 100 mgL^−1^ AgNPs

## Data Availability

All data collected or analyzed during this research are included in this article.
