# Correction: Umair Raza et al. Phytomediated Silver Nanoparticles (AgNPs) Embellish Antioxidant Defense System, Ameliorating HLB-Diseased ‘Kinnow’ Mandarin Plants. *Molecules* 2023, *28*, 2044

**DOI:** 10.3390/molecules31183174

**Published:** 2026-09-10

**Authors:** Muhammad Umair Raza, Fozia Abasi, Muhammad Shahbaz, Maria Ehsan, Wajiha Seerat, Abida Akram, Naveed Iqbal Raja, Zia-ur-Rehman Mashwani, Hammad Ul Hassan, Jarosław Proćków

**Affiliations:** 1 Department of Botany, PMAS-Arid Agriculture University, Rawalpindi 46300, Pakistan; mumairraza71@gmail.com (M.U.R.); abasifozia@gmail.com (F.A.); muhammad786943@gmail.com (M.S.); 14-arid-4355@student.uaar.edu.pk (M.E.); wajihaseerat@gmail.com (W.S.); abidaakram@uaar.edu.pk (A.A.); zia.botany@gmail.com (Z.-u.-R.M.); hammadulhassan883@gmail.com (H.U.H.); 2 Department of Plant Biology, Institute of Environmental Biology, Wrocław University of Environmental and Life Sciences, Kożuchowska 5b, 51-631 Wrocław, Poland

**Error in Figure**

In the original publication [1], there was a mistake in Figure 5. Upon careful review, the authors identified that an incorrect FTIR image had been inadvertently included during manuscript preparation. The correct Figure 5 is provided below:

**Figure Legend**

In the original publication, Figure 6 caption has a typo and broken grammar. The correct caption appears below.

**Figure 6.** Differential effects of plant-based silver nanoparticles on (**A**) chl a, (**B**) chl b, (**C**) total chl, (**D**) carotenoid content, (**E**) RWC, and (**F**) MSI. (“a” denotes the highest value, “b” the second highest, and so on).

**Typo Error in Abstract**

A correction has been made to Abstract: “phylogenic silver nanoparticles (AgNPs)” should be revised as “phytogenic silver nanoparticles (AgNPs)”.

**Text Correction**

A correction has been made to the fourth sentence in the Section 1. Introduction. The corrected sentence should be: “The family Rutaceae comprises almost 150 genera and 1600 species [4], with the most commercially important being mandarin Citrus reticulata Blanco [5].”

A correction has been made to Section 2.1, fourth paragraph, third sentence: “N–O stretching vibration” should be “C–N stretching vibration”; Ref. 54 should thus be removed from this phrase.

“The source of these absorption peaks was determined to be the O–H stretching vibration (3419 cm^−1^) in alcohol, phenol, and flavonoid compounds [50], C–H stretching vibration (2922 cm^−1^) of aromatic compounds [51], C=O stretching vibration (2370, 1734 cm^−1^) for carbonyl compounds [52], C=C stretching vibration (1637 cm^−1^) of aromatic compounds [53], N–O stretching vibration (1508 cm^−1^) in aliphatic amines [54], C–H stretching vibration (1458 cm^−1^) in methylene moieties [55], C–O stretching vibration (1261, 1097 cm^−1^) in alcohols and phenols [56], C=C stretching vibration (796 cm^−1^) of aromatic compounds, and, finally [57], C–Br and C–Cl bonding vibration (619, 453 cm^−1^, respectively) in organic compounds.” should be:

“The source of these absorption peaks was determined to be the O–H stretching vibration (3419 cm^−1^) in alcohol, phenol, and flavonoid compounds [51], C–H stretching vibration (2922 cm^−1^) of aromatic compounds [52], C=O stretching vibration (2370, 1734 cm^−1^) for carbonyl compounds [53], C=C stretching vibration (1637 cm^−1^) of aromatic compounds [54], C–N stretching vibration (1508 cm^−1^) in aliphatic amines, C–H stretching vibration (1458 cm^−1^) in methylene moieties [55], C–O stretching vibration (1261, 1097 cm^−1^) in alcohols and phenols [56], C=C stretching vibration (796 cm^−1^) of aromatic compounds, and, finally [57], C–Br and C–Cl bonding vibration (619, 453 cm^−1^, respectively) in organic compounds.”

A duplicate sentence in the Section 2.3, second paragraph, seventh sentence should be removed: “The best results were obtained with AgNPs at 75 mgL^−1^, which increased SSC by 74.75%.”

A correction has additionally been made to Section 3.2, Characterization of Nanoparticles, to include comprehensive details of all characterization techniques; the instruments used; and the respective institutions where the analyses were carried out, including the characterization performed at the Institute of Space Technology (IST). These additions ensure that the methodology is fully documented and that the origin of all experimental data is clearly identified. The corrected Section 3.2 should be:

“The green synthesis of AgNPs was confirmed by UV–Visible spectrophotometry (UV-752PC spectrophotometer, Shanghai Spectrum Instruments Co., Ltd., Shanghai, China, wavelength: 300–800 nm) at the Centralized Laboratory, PMAS-Arid Agriculture University, Rawalpindi, Pakistan. Structural characterization was conducted via scanning electron microscopy (TESCAN MIRA3 FEG-SEM, NanoImages, LLC, CA, USA) at the Institute of Space Technology (IST), Islamabad, Pakistan. Elemental composition was further analyzed using energy-dispersive X-ray spectroscopy (EDX; X-Max detector, Oxford Instruments, Abingdon, UK; AZtec software) at IST. Fourier transform infrared (FTIR) spectroscopy (Perkin-Elmer FTIR Spectrum, Akron, Ohio, USA) was carried out at Fatima Jinnah Women University, Rawalpindi, Pakistan, within the IR range of 500–4000 cm^−1^.”

**References Correction**

The original Ref. 54 should be moved to the legend of Figure 1 as Ref. 46. The correct legend appears below.

“**Figure 1.** Schematic illustration of the potential of AgNPs. AgNPs can be used in household items, food packaging, textiles, cosmetics, the main components of medicines, and antiseptics in medical equipment [39–41]. AgNPs have been used to stimulate growth in plants [42] and aid in fruit ripening [38]; have been developed as fungicides to prevent fungal infections [39]; and possess antidiabetic [43] and antibacterial potential [44–46], antiviral capabilities [43], and anticancer potential [44]. The activity of AgNPs against the causal agent of HLB ‘Candidatus Liberibacter asiaticus’ is also studied [47].”

The original Reference 99 has been added to the fourth paragraph in Section 2.2 Physiological Parameters as Ref. 64, where it supports the discussion on the regulation of soil bacterial diversity. The correct paragraph appears below. 

“In addition, an earlier study revealed that silver could speed up chlorophyll biosynthesis by easing the electron transport chain and the respiratory process. As a result, a rise in chlorophyll content in HLB-infected plants treated with AgNPs could help to restore the photosynthetic machinery and thus growth qualities. Furthermore, devastating effects on the stroma and grana lamellae are avoided by silver nanoparticles; AgNPs also help chloroplasts to protect chloroplast enzymes and regulate soil bacterial diversity [64], thus speeding up the photosynthetic machinery’s biosynthesis [65].”

The original Ref 99 has been corrected and replaced with the original Reference 105 as Ref 100 in the last paragraph of Section 2.4. Fruit Quality Parameters. The correct paragraph appears below.

“All monosaccharides and disaccharides found in food, regardless of their source, constitute total sugars (TS). The term “sugar” normally refers to sucrose (table sugar), although it can also refer to all sugars [100]. Sugars are the main source of energy and structural material for plant defense responses and may also act as signal molecules that interact with the hormone signaling network of the plant immune system [101]. The TS level of HLB-infected ‘Kinnow’ mandarin plants was considerably lower than that of healthy citrus plants [102]. Exogenous foliar sprays of green-synthesized AgNPs increased the TS levels in ‘Kinnow’ plants infected with HLB, helping them to obtain higher taste quality (Figure 9). Using AgNPs at a dose of 75 mgL^−1^ yielded highly effective results, according to this study. The findings of this study are comparable to those of Faghihi.”

The original References 104 and 105 have been removed from Section 3.1.2, and the appropriate citations, originally Refs 101 and 103, have been inserted as Refs 102 and 104 for the described methodology. The correct paragraph appears below.

“Approximately 50 mL of *Moringa oleifera* aqueous leaf extract was poured dropwise into 450 mL of silver nitrate solution (5 mM) and the mixture was stirred continuously for 4 h at room temperature for complete AgNP biosynthesis. Color changes of reaction mixtures were closely monitored to confirm nanoparticle formation; AgNP production was characterized by a dark brown color of the reaction solution [104]. After the formation of AgNPs, the solution was stored in the dark at room temperature to prevent the agglomeration of AgNPs in the solution. Subsequently, the solution was centrifuged at 10,000 rpm for 15 min at 8 °C to collect pure AgNPs, the supernatant was collected, and purified AgNPs were oven-dried at 80 °C overnight for UV–vis analysis [102,104] (Figure 10).”

The original Reference 104 has been reassigned to its appropriate location as Ref 115 in Section 3.4.7 (Proline Content and Sugar Content).

“3.4.7. Proline Content and Sugar Content

The proline content of the treated and untreated leaves of the ‘Kinnow’ mandarin plants was measured at 520 nm following the method published by Kunwar et al. [114] and Monreal et al. [115]. For sugar content, absorbance was observed at a 490 nm wavelength, adopting the procedure of Thompson et al. [116].”

With this correction, the order of some references has been adjusted accordingly. The authors state that the scientific conclusions are unaffected. This correction was approved by the Academic Editor. The original publication has also been updated.

## Figures and Tables

**Figure 5:**
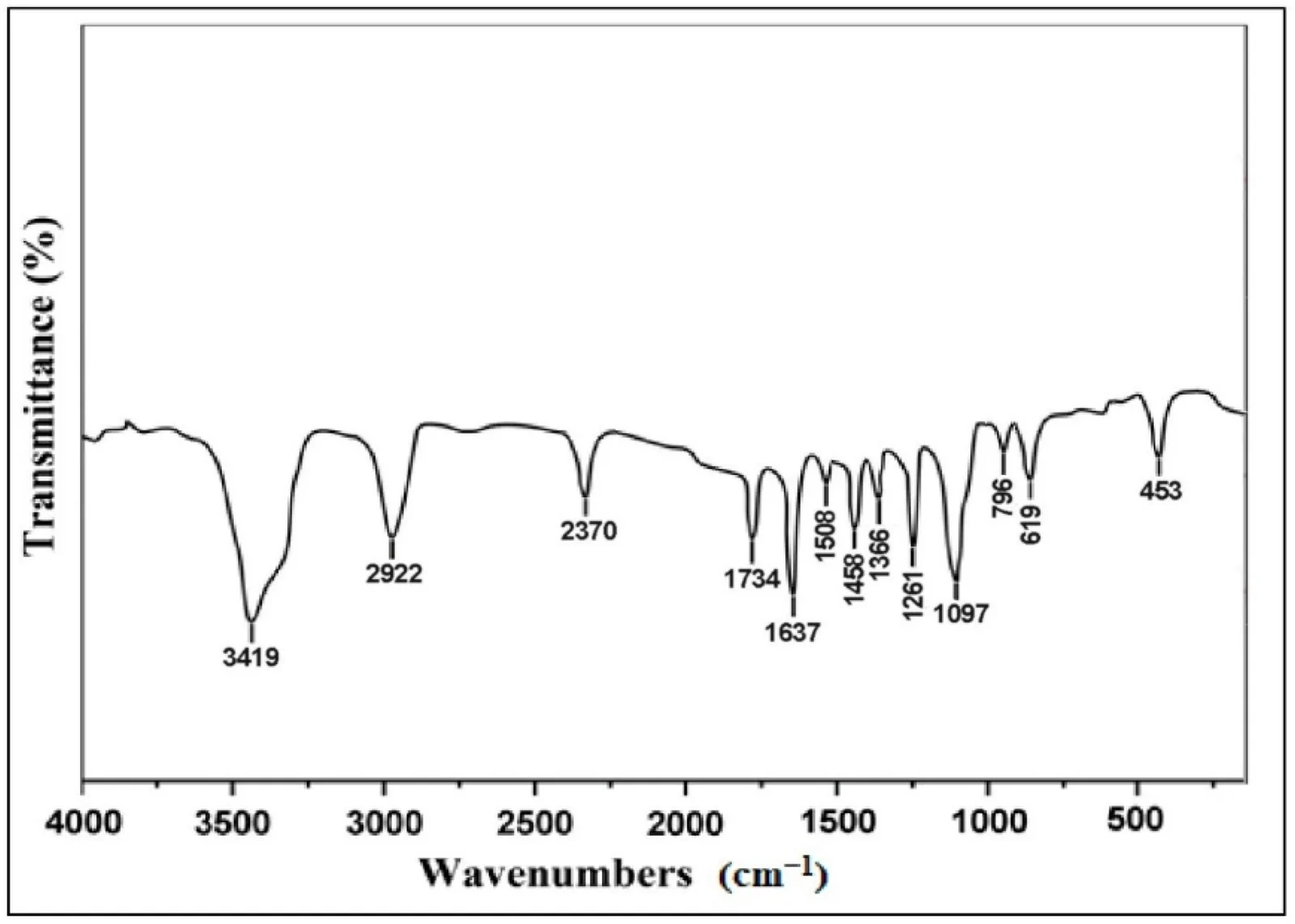
FTIR of AgNPs.
